# The metabolic-epigenetic interface: lysine succinylation orchestrates bidirectional crosstalk in neurodegenerative disorders

**DOI:** 10.3389/fneur.2025.1678595

**Published:** 2025-12-16

**Authors:** Fanglei Chai, Chong Liu, Yandong Liu, Wei Zou

**Affiliations:** 1 Heilongjiang University of Chinese Medicine, Harbin, Heilongjiang, China; 2 Second Affiliated Hospital of Heilongjiang University of Chinese Medicine, Harbin, Heilongjiang, China; 3 Department of Integrated Traditional Chinese and Western Medicine, Heilongjiang Provincial Hospital, Harbin, Heilongjiang, China; 4 First Affiliated Hospital, Heilongjiang University of Chinese Medicine, Harbin, Heilongjiang, China

**Keywords:** metabolic-epigenetic crosstalk, mitochondria, NDD, SIRT5, succinylation

## Abstract

Succinylation, a nexus between metabolism and epigenetic regulation, is a central factor in the onset and progression of neurodegenerative diseases (NDDs). Research has demonstrated a close association between NDDs and neuronal metabolic disorders. Succinylation regulates the interaction between energy metabolism and epigenetic networks, establishing the pathological mechanism of “metabolic-epigenetic bidirectional regulation.” In metabolic stress, such as mitochondrial dysfunction or enhanced glycolysis, succinyl-CoA increases, causing uncontrolled succinylation. These modifications impair the function of proteins associated with synaptic plasticity, leading to disorders in synaptic transmission and neuronal damage. Concurrently, succinylation regulates the activity of enzymes involved in DNA methylation and epigenetic reprogramming, impairing neuronal recovery and creating a vicious cycle. This regulatory network displays bidirectional self-reinforcing characteristics. Metabolic disorders influence epigenetic states through succinylation. Epigenetic abnormalities inhibit the transcription of genes associated with mitochondrial metabolism, exacerbating energy metabolism defects and oxidative stress. This leads to irreversible degenerative changes in neurons. At the therapeutic level, targeting succinylation can disrupt the metabolic-epigenetic pathological loop and restore synaptic function. In short, understanding how succinylation is regulated may lead to new treatment options for neurodegenerative diseases.

## 1 Introduction

Neurodegenerative diseases (NDDs) are characterised by a progressive loss of neuronal function, with their onset and progression being profoundly influenced by metabolic dysregulation. Impaired neuronal energy metabolism, characterised by insufficient ATP production and mitochondrial dysfunction, can result in synaptic dysfunction and accelerate degenerative changes (1). During the process of aging, an accumulation of reactive oxygen species (ROS) further exacerbates DNA damage and mitochondrial dysfunction, thereby creating a vicious cycle (2). Moreover, the combined effects of genetic susceptibility and environmental exposure on neurons within an aging context form the pathological basis for the vast majority of sporadic neurodegenerative diseases, including Alzheimer’s disease (AD) and Parkinson’s disease (PD).

Epigenetic mechanisms regulate gene expression related to neuronal survival, plasticity, and repair through pathways such as DNA methylation, histone modifications, and non-coding RNAs, without altering DNA sequences (3). Under healthy conditions, epigenetic networks coordinate synaptic function and cellular homeostasis. However, the progression of neurodegenerative diseases can be exacerbated by disruptive factors, such as environmental toxins, which have the capacity to impair this regulatory system (4). Conventionally, metabolic and epigenetic systems were regarded as being functionally and regulatorily independent: metabolism was understood to govern energy supply and biosynthesis, while epigenetics was considered to govern chromatin structure and gene programming. Recent studies have demonstrated that metabolic intermediates can function as substrates or regulators for epigenetic modifications, thereby directly participating in processes such as histone modifications and DNA methylation. This observation suggests the formation of a “metabolism-epigenetics” regulatory network. Within this framework, intermediate metabolites accumulated due to metabolic abnormalities can influence enzyme activity, protein structure, and signal transduction through post-translational modifications (PTMs), thereby reshaping epigenetic states. Consequently, metabolite-mediated PTMs serve as a crucial bridge connecting metabolomics with proteomics/epigenomics.

Recent studies have demonstrated that intermediates in the tricarboxylic acid (TCA) cycle possess “non-metabolic functions” and can directly serve as substrates, cofactors, or competitive inhibitors for epigenetic modifications. They participate directly in the construction of the “metabolism-epigenetics” interaction network. Acetyl-CoA, a pivotal node product of glycolysis and fatty acid *β*-oxidation, serves not only as a core intermediate in energy metabolism but also as an acetyl donor. It has been demonstrated that this protein facilitates the process of histone acetylation through its interaction with histone acetyltransferases (5). This process neutralizes the positive charge of lysine residues, thereby weakening interactions between histones and DNA. Concurrently, this process promotes chromatin structural relaxation and activates gene transcription (6, 7). S-adenosylmethionine (SAM) is a critical methyl donor that plays a pivotal role in the processes of histone and DNA methylation modifications. Within the cytoplasm, the folate cycle and methionine cycle work in concert to ensure the maintenance of SAM levels. Additionally, mitochondrial one-carbon metabolism plays a regulatory role in its homeostasis (8). Research indicates that SAM transporter deficiency in *Slc25a26* knockout models disrupts mitochondrial energy metabolism, suggesting a potential regulatory role of SAM in metabolic processes through mitochondrial methylation. However, the direct impact of SAM on nuclear epigenetic mechanisms remains to be fully validated (9).

*α*-Ketoglutaric acid (α-KG) functions not only as an intermediate in the TCA cycle but also as a substrate for histone demethylases. In the presence of Fe^2+^ and O₂, *α*-KG has been observed to promote histone demethylation while generating succinate and CO₂, thereby maintaining gene activation states (10). Conversely, succinate and fumarate competitively inhibit *α*-KG-dependent enzymes (e.g., TET/JMJD), leading to increased DNA methylation and decreased histone acetylation levels. This results in the compaction of the chromatin structure and the subsequent impediment of transcription (11, 12). Furthermore, succinate functions as a direct donor of succinyl-CoA for histone modification, thereby amplifying the epigenetic regulatory effect.

Fumaric acid plays a dual role in mitochondrial metabolism, and its abnormal accumulation can induce KEAP1 protein succinylation, activate the Nrf2 pathway, and sustain the expression of antioxidant genes. Research has demonstrated that fumarate hydratase deficiency results in mitochondrial network remodeling and mtDNA release, consequently activating the innate immune response (13, 14). These findings reveal the pivotal role of metabolites in linking energy metabolism, epigenetic regulation, and cellular stress responses.

Research into the pathogenesis of neurodegenerative diseases has long grappled with the complex interplay between metabolic dysregulation and epigenetic imbalance. In recent years, the dynamic regulatory network of protein PTMs has provided a crucial breakthrough for unraveling this challenge. Among these, lysine succinylation has garnered significant attention due to its high sensitivity to metabolites and its pivotal role in the regulation of chromatin. This modification, utilizing succinyl-CoA as a donor, reversibly introduces negatively charged succinyl groups within mitochondria. It has been demonstrated that this substance exerts a direct regulatory effect on enzyme activity, remodels chromatin structure, and influences gene transcription networks. Consequently, it plays a role in determining cell fate (11, 15). A 2011 study was the first to identify three acyltransferase proteins involved in mitochondrial metabolism under native conditions, thereby revealing the fundamental role of this modification in metabolic regulation (16). Succinylation modulates metabolic enzyme activity and pathways in response to nutritional conditions. It also occurs on core histones, primarily enriched in mitochondrial metabolic processes, exerting critical effects on gene expression and DNA repair (17).

The brain is a highly metabolically active organ, and energy dysregulation in the brain is closely associated with various central nervous system disorders. According to the extant research, *α*-ketoglutarate dehydrogenase (α-KGDH) has been shown to promote succinylation by increasing succinyl-CoA, which is derived from propionate or ketone body metabolism in neurons. This, in turn, has been demonstrated to accelerate neurodegenerative processes (18). In neurons, mitochondrial succinylation levels exhibit dynamic fluctuations in response to metabolic states. Impairment of glycolysis, glutathione depletion, disruption of the TCA cycle, dysfunction of the electron transport chain, and decoupler use have been observed to result in a reduction of succinylation levels (17).

**Figure 1:**
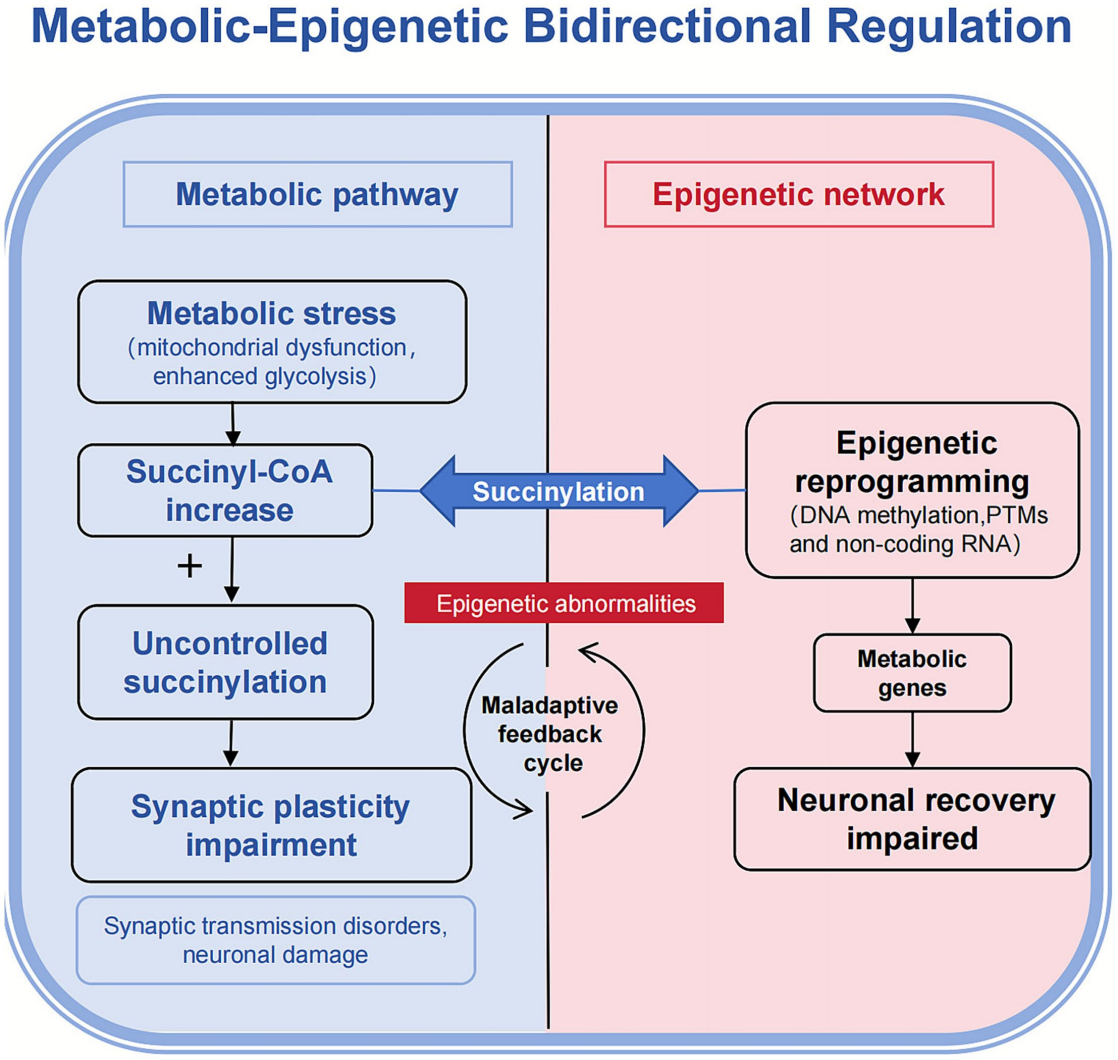
The succinate-mediated bidirectional metabolic-epigenetic coupling hypothesis. This schematic illustrates the succinylation bidirectional regulatory network hypothesis. Energy metabolism abnormalities in neurodegenerative diseases regulate succinylation levels, which then actively disrupt synaptic plasticity, mitochondrial quality control, and epigenetic programming. These disruptions form a pathological cycle where ensuing epigenetic abnormalities (e.g., aberrant DNA methylation and non-coding RNA dysregulation) further suppress metabolic gene expression, thereby sustaining and amplifying the initial metabolic stress.

These findings prompt the following considerations: The present study hypothesises that succinylation constitutes a molecular hinge between metabolic homeostasis and epigenetic regulation, driving neurodegenerative cascades by establishing bidirectional signalling networks. Current evidence indicates that metabolic microenvironmental disturbances – such as abnormalities in the TCA cycle, impaired glycolysis, and disrupted electron transport chains – can alter succinyl-CoA concentrations, thereby influencing succinylation levels (17). Conversely, histone succinylation (e.g., H3K79succ) has been demonstrated to regulate nucleosome stability and chromatin dynamics (19). This finding suggests that succinylation does not merely passively respond to metabolic fluctuations, but also actively contributes to the reshaping of the epigenetic landscape, thereby establishing bidirectional regulatory circuits. However, extant studies predominantly focus on the unidirectional regulatory effects of succinylation, lacking systematic analysis of its integration mechanisms within metabolic-epigenetic interaction networks. This review proposes the succinylation bidirectional regulatory network hypothesis: in the process of neurodegenerative disease, energy metabolism abnormalities regulate succinylation levels by altering metabolic flux, inducing functional modifications to neuronal proteins. These changes make neural decline worse by disrupting synaptic plasticity, mitochondrial quality control, and epigenetic reprogramming. Abnormal DNA methylation and dysregulation of non-coding RNA form a pathological cycle regulating the expression of metabolism-related genes (Figure 1).

This review will discuss the chemical biology of succinylation modification and its regulatory mechanism, the dual regulatory roles of this modification in neurodegenerative diseases, and the challenges and translational perspectives of key node targeting strategies based on bi-directional networks.

## 2 The regulation of protein succinylation is influenced by metabolite levels

Lysine succinylation, a type of PTM, is defined as the covalent linkage of succinyl groups to the lysine residues of substrate proteins through donors. In comparison with methylation and acetylation, succinylation modifications introduce larger, negatively charged functional groups, thereby more significantly altering protein conformation and functional properties. Recent studies have substantiated the existence of a dual-drive mechanism for succinylation, encompassing both enzymatic catalysis and non-enzymatic reactions.

**Figure 2:**
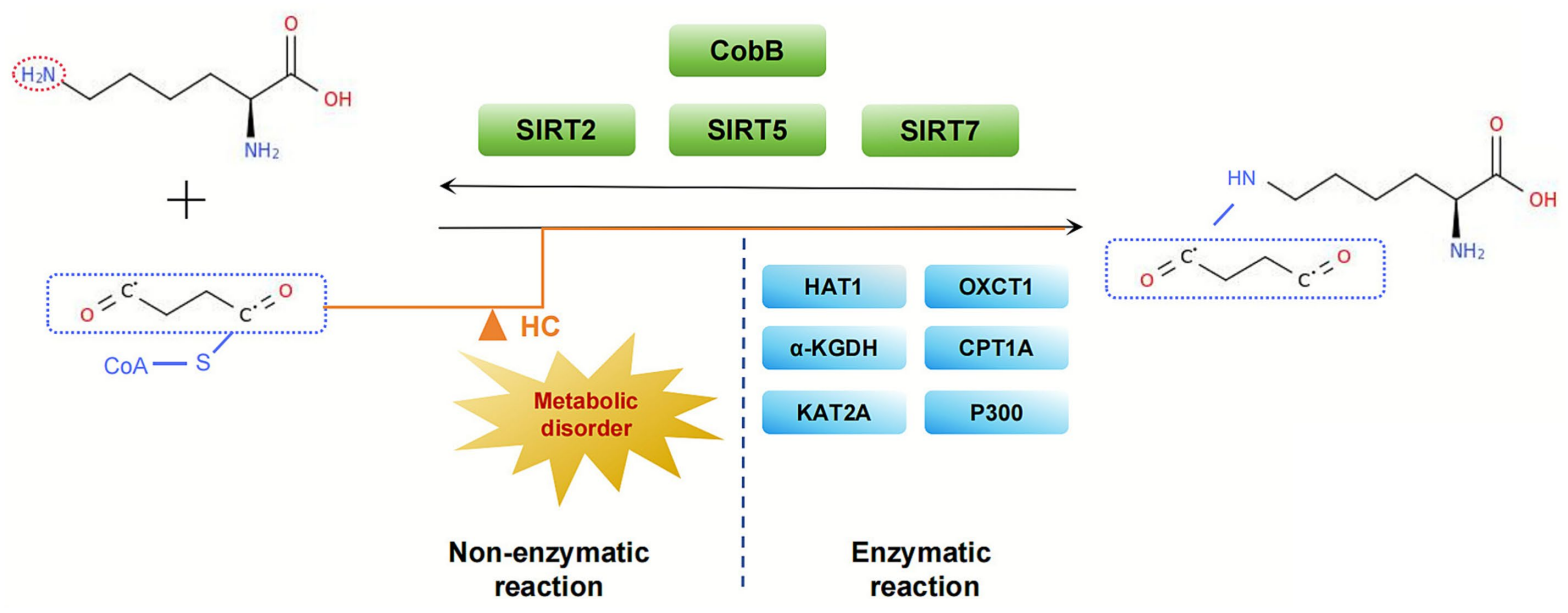
Enzymatic and non-enzymatic pathways of lysine succinylation. This illustration depicts the dual mechanisms governing lysine succinylation. The enzymaticpathway involves succinyltransferases (e.g., KAT2A) and desuccinylases (e.g., SIRT5), which utilize succinyl-CoA from mitochondrial metabolism. Concurrently, elevated levels of succinyl-CoA can promote non-enzymatic, spontaneous succinylation, thereby contributing to global modification levels.

In the enzymatic pathway, succinylation levels are jointly regulated by succinyl-CoA donors, succinyl transferases, and desuccinylases. Succinyl-CoA can be derived from amino acid metabolism or the TCA cycle. Intracellular lysine succinylation is catalyzed by succinyl transferases such as OXCT1, KAT2A, HAT1/CPT1A (11, 20–23). Concurrently, desamoylases CobB and SIRT2/5/7 maintain the dynamic equilibrium of this modification by removing the succinyl group (24). In non-enzymatic pathways, succinyl-CoA directly drives spontaneous modification of lysine residues through its chemical reactivity. According to the extant data, succinyl-CoA has been demonstrated to directly modify lysine residues *in vitro*. Furthermore, the levels of succinylation in yeast cells are globally regulated by succinyl-CoA metabolism. This finding indicates that metabolite concentration-driven non-enzymatic reactions are a significant formation mechanism for succinylation modification [Figure 2; Weinert et al. (25)].

The TCA cycle is the central metabolic hub within eukaryotic mitochondria. It serves as the ultimate common pathway for the oxidation of carbohydrates, proteins, and lipids. It plays a vital role in key biological processes such as gluconeogenesis, amino acid metabolism, and lipid synthesis (26). Within this cycle, isocitrate is catalyzed by isocitrate dehydrogenase (IDH) to produce *α*-KG and NADH. α-KG is subsequently converted to succinyl-CoA and releases NADH through oxidative decarboxylation by the α-KGDH (27). Subsequently, succinyl-CoA undergoes substrate-level phosphorylation by succinyl-CoA synthetase to form succinate.

**Figure 3:**
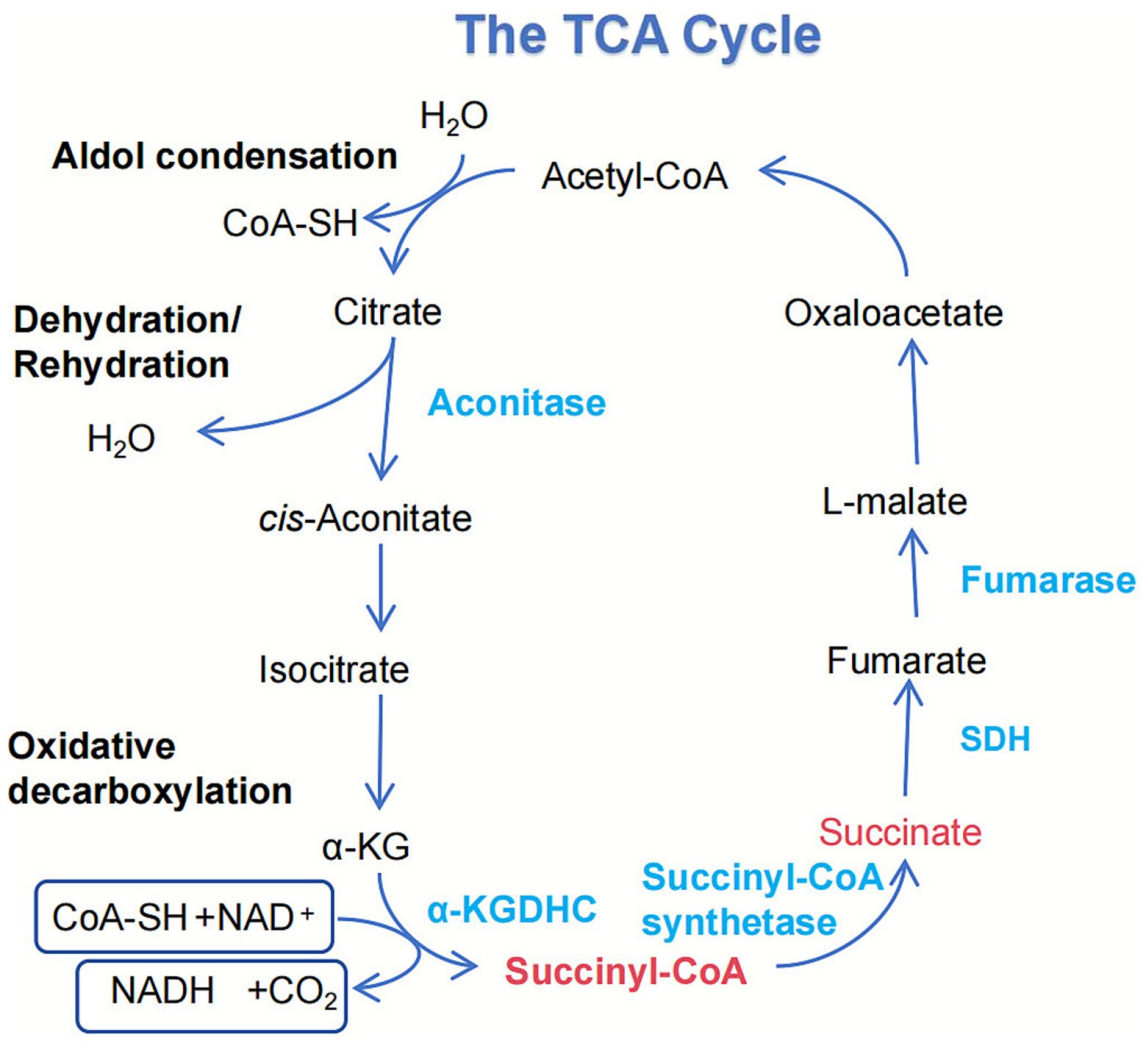
Integration of succinate metabolism and its dynamic regulation. This schematic details the generation of succinate within the mitochondrial TCA cycle. Succinate is primarily produced through the oxidative decarboxylation of α-ketoglutarate, a reaction catalyzed by the α-KDHC. Additionally, it is formed from succinyl-CoA by succinate-CoA ligase, a reaction that concurrently drives substrate-level phosphorylation to generate GTP. As a central metabolite, succinate is subsequently oxidized to fumarate by SDH.

Succinate concentration is a key component of the TCA cycle, and its regulation is a dynamic process involving multiple metabolic activities, including glucose breakdown, fatty acid oxidation, and glutamine metabolism. Concurrently, epigenetic mechanisms such as DNA methylation and histone modifications indirectly influence succinate production rates by regulating the expression of TCA cycle-related genes (e.g., SDHA, SDHB). In pathological conditions marked by an imbalance between energy demand and oxygen supply, such as ischemia and hypoxic exercise, significant accumulation of succinate has been observed within mitochondria and in the intracellular/extracellular environment (28, 29). In addition to the direct reduction of succinate dehydrogenase (SDH) activity leading to succinate accumulation, metabolic pathways such as the malate–aspartate shuttle and purine nucleotide cycle can also convert fumarate to succinate through a process of reverse catalysis (30–32). Furthermore, the *γ*-aminobutyric acid (GABA) pathway intersects with the TCA cycle, ultimately yielding succinate and expanding its diverse metabolic origins (Figure 3).

The cross-cell transport mechanism of succinate involves metabolic coupling between neurons and glial cells, as well as exosome-mediated signaling. Research has demonstrated that succinate can be transferred between cells via specific transporters, including members of the SLC13 family. *SLC13A3* has been identified as a regulator of succinate uptake in the kidney (33). Furthermore, it has been demonstrated that exosome—which are pivotal in intercellular communication—have been found to transport essential components of the TCA cycle and glycolysis (e.g., PDHA1 and *α*-KG). This finding suggests the possibility that succinate may utilize analogous extracellular transport pathways (34, 35).

Succinate fulfills a signaling role and exhibits triple regulatory functions. Firstly, it functions as an intermediate metabolite, contributing to cellular energy metabolism and respiratory regulation. Secondly, it serves as a substrate for succinyl-CoA, playing a role in post-translational protein regulation through succinylation modification. Thirdly, it can act as a hormone-like molecule, activating succinate receptors and initiating downstream signaling pathways. Molnár et al. (36) revealed via calcium imaging that succinate independently induces calcium transients in glial cells, independent of neuronal activity. This finding suggests that succinate plays a distinctive role in energy metabolism and calcium signaling regulation, thereby emphasizing the function of glial cells in metabolic-signaling coupling within the nervous system (36). Recent studies have revealed that succinate regulates the stability of the transcription factor FOXP3 via a “succinylation-ubiquitination” molecular switch. In patients diagnosed with inflammatory bowel disease (IBD), plasma succinate levels exhibited a significant negative correlation with FOXP3 expression. During disease remission, as succinate levels decreased, Treg cell function gradually recovered, suggesting that metabolites precisely regulate immune cell function through PTMs (37). Lu et al. (38) further revealed a dual mechanism of succinate in tumor immune regulation. The employment of electroporation technology to encapsulate succinate within tumor-derived microparticles (SMPs) facilitated its precise delivery to the mitochondria and nuclei of tumor-associated macrophages (TAMs). Within the mitochondrial matrix, succinate instigates a process of succinylation of IDH2, leading to the inhibition of its enzymatic activity. Consequently, this results in the enhancement of glycolysis and the suppression of the TCA cycle. This process leads to the conversion of metabolic intermediates into L-2-hydroxyglutarate (L-2HG), thereby stabilizing HIF-1α and activating inflammatory signaling. Within the nucleus, succinate specifically succinylates histone H3K122, accumulating at the lactate dehydrogenase A (LDHA) gene promoter region to enhance its expression (38). This metabolic reprogramming has been shown to drive TAMs to polarize from an M2 immunosuppressive phenotype toward an M1 antitumor phenotype. This offers a novel strategy for tumor immunotherapy.

The mechanisms that underlie the interplay between metabolism and epigenetics are being elucidated through scientific inquiry. Liu et al. (39) demonstrated the existence of a non-canonical TCA within the cell nucleus, where citrate synthase (CS) influences histone acetylation by regulating nuclear acetyl-CoA levels, directly linking transcription and epigenetic regulation. Conversely, the dysfunction of SDH has been shown to disrupt the process of chromatin succinylation, impede the capacity for DNA repair, and augment sensitivity to genotoxic drugs (40). Willnow et al. further revealed that the process of fatty acid *β*-oxidation supplies a carbon source for histone acetylation, and its inhibition leads to a reduction in H3K18ac levels in the proximity of developmental genes. This finding suggests that metabolic disorders may influence key gene activity through epigenetic dysregulation (41). In summary, metabolic states exert fine-tuned regulatory effects on genome-wide transcriptional control and DNA repair through mechanisms such as histone post-translational modifications. As a metabolism-sensitive modification, succinylation likely serves a bridging role analogous to acetylation within the metabolic-epigenetic interaction network, translating cellular metabolic states into heritable epigenetic information.

Notably, astrocytes play a pivotal role in metabolic reprogramming. For instance, the absence of SIRT3 among Class III histone deacetylases (Sirtuins) prompts a shift in the microenvironment of neovascularization from fatty acid oxidation to glycolysis, thereby enhancing vascular endothelial growth factor expression in astrocytes. This promotes the transition of pathological vasculature toward a physiological phenotype, illustrating a crucial mechanism by which glial cells support neuronal function through metabolic regulation (42). From a regulatory network perspective, epigenetic mechanisms indirectly influence the dynamic equilibrium of succinate concentration by modulating the expression of metabolism-related genes. Succinate levels are directly maintained by metabolic cycles, however, they may be profoundly affected by epigenetically regulated metabolic genes. This forms a complete regulatory circuit from epigenetics to metabolic state, and back to cellular function (43).

Nicotinamide adenine dinucleotide (NAD) is a core coenzyme in cellular energy metabolism. It relies on the dynamic equilibrium of its oxidized form (NAD+) levels and the NAD+/NADH ratio to maintain cellular redox homeostasis (44–47). At the epigenetic level, NAD + has been shown to play a role in the fine-tuning of neural function by influencing the activity of Sirtuins. SIRT5, a mitochondrial-localized desulfatase, plays a unique role in maintaining energy homeostasis by catalyzing desulfation reactions to preserve the activity of ATP synthase and CS (48, 49). Research has demonstrated that SIRT5 deficiency results in increased succinylation levels in CS and ATP synthase, thereby impeding their enzymatic activity and disrupting the TCA cycle and ATP synthesis. Conversely, SIRT5-mediated desuccinylation has been shown to effectively alleviate mitochondrial metabolic dysfunction in pathological states, such as subarachnoid hemorrhage (50). NAD + has been demonstrated to participate in cellular stress responses through its regulatory impact on Sirtuin activity. Additionally, its role extends to the formation of a dynamic regulatory network, where it influences levels of succinylation and the formation of metabolic intermediates, such as succinate.

Accordingly, succinylation manifests a dualistic mechanism in the context of epigenetic regulation, encompassing both direct epigenetic modification and indirect epigenetic effects through metabolic reprogramming. It has been demonstrated that metabolic states exert fine-tuned regulatory effects on genome-wide transcriptional control and DNA repair through mechanisms such as histone PTMs. Succinylation, a metabolism-sensitive modification, functions as a pivotal bridge within the metabolic-epigenetic interaction network. This network plays an integrative role in maintaining mitochondrial function, energy homeostasis, and neuroprotection.

## 3 The dynamic balance of metabolic homeostasis is regulated by epigenetic-metabolic networks

Epigenetic regulation constitutes a core mechanism for dynamically modulating gene expression and influencing cellular phenotypes through reversible chemical modifications without altering DNA sequences. Genetic modifications serve as a pivotal element of epigenetic regulation, meticulously orchestrating the activation and silencing states of genes through DNA methylation, histone PTMs, and non-coding RNA-mediated chromatin remodeling. This intricate process establishes heritable transcriptional programming patterns, shaping the genetic blueprint of an organism’s development and heredity.

### 3.1 The immediate consequences of succinylation on epigenetic inheritance

#### 3.1.1 Crosstalk between succinylation and other types of PTMs

Acylation modifications are defined by their heterogeneity, dynamism, and synergistic effects, and they play pivotal regulatory roles in pivotal biological processes such as central metabolism, protein synthesis, and chromosome segregation. The molecular mechanisms underlying these effects have been systematically validated in bacterial models. Histone succinylation, a significant type of acylation modification, has been demonstrated to play a potential role in the regulation of gene expression. A comparative analysis of the functions of the deacetylase ScCobB1 and desuccinylase ScCobB2 in *Streptomyces indigoferus* was conducted by Yang et al. (51). This analysis was complemented by a comprehensive proteomic investigation of PTMs, which resulted in the revelation of a global competitive relationship between acetylation and succinylation at lysine sites. Furthermore, evidence was presented demonstrating that these modifications synergistically regulate multiple core metabolic pathways (51). Moreover, a reciprocal relationship has been observed between succinylation and phosphorylation, suggesting a complex interplay among these PTMs. Research indicates that the process of phosphorylation indirectly regulates succinylation through alterations to the microenvironment of metabolites such as succinyl-CoA. Guo et al. (52) conducted a combined analysis of the succinylation and phosphorylation profiles in lung cancer patients, revealing that histone deacetylase (HDAC) phosphorylation enhances its deacetylase activity, thereby promoting mitochondrial protein succinylation levels. This cascade regulatory mechanism impacts metabolic reprogramming in cancer cells and offers novel targeted strategies for intervening in mitochondrial succinylation. Furthermore, ubiquitination—a pivotal modification that regulates protein degradation—may indirectly influence the accessibility of modification sites like succinylation by altering subunit conformation or charge distribution. Tsimokha et al. (53) discovered that specific sites on proteasome subunits simultaneously bear acetylation, ubiquitination, and succinylation modifications, suggesting a complex network of interactions among multiple PTMs that collectively regulate proteasome function.

Collectively, these findings reveal that succinylation forms competitive, synergistic, or cascading relationships with modifications such as acetylation, phosphorylation, and ubiquitination. This construction establishes a multi-level, dynamically interconnected cellular regulatory system.

#### 3.1.2 Competition of histone modifications

Histone succinylation, as a crucial epigenetic modification, directly participates in gene expression regulation by altering chromatin spatial conformation and recruiting specific regulatory factors. Li et al. (19) conducted a systematic investigation into the role of the desuccinylase SIRT7 in DNA double-strand break (DSB) repair using a U2OS cell model lacking SIRT7. Laser microirradiation and immunofluorescence analysis revealed that SIRT7 rapidly recruits to DSB sites and catalyzes H3K122 desamidation in a PARP1-dependent manner. Mechanistically, H3K122 succinylation introduces negative charges that neutralize the positive charge of histones, thereby weakening their interaction with the DNA phosphate backbone and resulting in chromatin relaxation. Conversely, SIRT7-mediated desuccinylation promotes chromatin repackaging, thereby ensuring efficient DSB repair. At the nucleosome structural level, Jing et al. (54) utilized expression protein ligation (EPL) technology to synthesize site-specific succinylated histone H4 (H4K77succ) and reconstituted mononucleosomes *in vitro*. Electrophoretic mobility and Förster resonance energy transfer (FRET) analyses revealed that H4K77succ significantly reduced nucleosome stability, promoted DNA unwinding, and facilitated access of regulatory proteins, such as transcription factors, to previously shielded DNA regions. This modification also accelerated nucleosome unwinding kinetics, thereby further confirming succinylation’s pivotal role in the regulation of chromatin dynamics (54).

In summary, lysine succinylation achieves precise regulation of protein function and chromatin state at the molecular level through charge modulation, spatial conformation remodeling, and cross-interactions with other modifications. These findings confirm succinylation’s essential role as a novel histone modification in maintaining genomic stability and precise transcriptional regulation.

#### 3.1.3 Epigenetic regulators

The process of DNA hypermethylation has been demonstrated to silence genes associated with the mitochondrial respiratory chain, leading to impaired oxidative phosphorylation and a cellular reliance on glycolysis for energy provision. While the direct regulatory relationship between succinylation and DNA methylation remains to be fully elucidated, it is noteworthy that the direct effects of other acylation modifications (e.g., histone acetylation) on the activity of DNA methylases (DNMTs) have been progressively revealed. While DNA methylation is a prevalent epigenetic signal that represses gene transcription, histone deacetylation has also been demonstrated to inhibit transcription. DNMT1, the key enzyme responsible for DNA methylation, has been identified as a critical regulator in this process. Peng et al. (55) discovered that SIRT1-mediated deacetylation dynamically regulates DNMT1 activity through domain-specific modifications. Deacetylation of lysine residues within the GKG linker region impairs its transcription repression capacity that is independent of methyltransferase activity, while acetylation levels at specific sites (K1349/K1415) in the catalytic domain directly modulate its enzymatic function. These findings suggest that acetylation modifications directly influence the establishment and maintenance of DNA methylation signals through an “epigenetic enzyme activity regulation axis,” providing a theoretical framework for exploring succinylation’s role in analogous networks. Succinylation may play a role in the functional regulation of DNMTs and other epigenetic regulatory elements through mechanisms that are distinct from one another, including competitive lysine modification and metabolite-dependent enzyme activity modulation. Consequently, these mechanisms contribute to the formation of an integrated regulatory pattern within the metabolism-epigenetics interaction network (55).

### 3.2 Epigenetic regulation of succinylation levels

Epigenetic mechanisms can indirectly regulate protein succinylation by modulating the activity of succinyl transferases. Using *in vitro* hepatocellular carcinoma cell models and nude mouse xenograft models, Fan et al. (56) systematically demonstrated that METTL14-mediated m6A methylation promotes the processing of pri-miR-122 into mature miR-122-5p, which in turn targets and suppresses the expression of the succinyl transferase KAT2A. As the primary enzyme for H3K79 succinylation, downregulation of KAT2A led to a global reduction in protein succinylation, including on *β*-catenin. The decrease in β-catenin succinylation enhanced its stability and consequently activated the Wnt/β-catenin signaling pathway. Thus, this work establishes a clear connection between RNA methylation (METTL14), microRNA regulation (miR-122-5p), and protein succinylation (KAT2A). These findings delineate a multi-level, cross-regulatory epigenetic axis, providing systematic experimental evidence for how diverse epigenetic mechanisms converge to regulate succinylation in pathological settings.

Non-coding RNAs function as pivotal mediators in metabolic-epigenetic interactions, serving as a bridge that integrates metabolic and epigenetic signals. At the level of upstream metabolic regulation, cellular metabolic states dynamically modulate histone modifications or transcription factor activity in specific promoter regions by altering metabolite concentrations. This, in turn, influences the transcriptional output of microRNAs (miRNAs) and long non-coding RNAs (lncRNAs). At the downstream epigenetic regulation level, non-coding RNAs achieve precise bidirectional control over the metabolic-epigenetic network by targeting the mRNAs of metabolic enzymes, succinyl-CoA transferase regulators, and associated epigenetic modification enzymes. It has been demonstrated that certain long non-coding RNAs (lncRNAs) possess the ability to directly recruit chromatin modification complexes to specific genomic sites. This process serves to guide the establishment of localized histone modifications or DNA methylation, thereby converting transient metabolic signals into stable epigenetic memory (57). Of particular note is the bidirectional regulatory circuit between histone succinylation and non-coding RNAs: On one hand, succinylation at key histone sites (e.g., H3K122) significantly weakens histone-DNA interactions by introducing negative charges and bulky groups, leading to chromatin relaxation and thereby activating gene transcription, including long non-coding RNAs and microRNAs. Conversely, non-coding RNAs have the capacity to exert an opposing influence on global protein succinylation levels by modulating the expression of succinylation-related enzymes, including KAT2A and SIRT5. This regulatory mechanism forms a negative feedback loop, thereby contributing to the maintenance of homeostatic balance in the succinylation response. This interaction mechanism is particularly crucial under metabolic stress conditions, participating in maintaining metabolic homeostasis by integrating nutritional signals with gene expression programs.

### 3.3 The bidirectional interaction between metabolic abnormalities and epigenetics

Epigenetic mechanisms have evolved to establish intimate associations with cellular metabolic states and pivotal enzyme activities by regulating the expression of metabolism-related genes. The activity of numerous chromatin-modifying enzymes is contingent on metabolic intermediates such as acetyl-CoA, *α*-ketoglutarate, S-adenosylmethionine, and NAD + as cofactors. Concurrently, succinylation modification itself dynamically regulates the structure and function of metabolic enzymes (58).

At the level of energy metabolism, typically differentiated cells primarily rely on mitochondrial oxidative phosphorylation for energy production. In contrast, tumor cells and stem cells tend to adopt the “Warburg effect” or Warburg-like metabolic patterns, favoring glycolysis over the TCA cycle (59). Research has demonstrated that succinylation plays a crucial role in preserving this metabolic phenotype, achieved through dynamic modifications of pivotal metabolic enzymes implicated in glycolysis, the pentose phosphate pathway, and the TCA cycle. In undifferentiated stem cells, elevated levels of succinylation have been shown to suppress TCA cycle enzyme activity, thereby prioritizing glycolysis for energy production. As differentiation progresses, the process of downregulation of succinylation facilitates the restoration of mitochondrial oxidative phosphorylation capacity, thereby providing the energy foundation for cellular maturation (12).

At the molecular level, succinylation has been shown to induce substantial alterations in the charge state and conformation of lysine residues, primarily through the introduction of large, strongly negatively charged groups. A multitude of studies have confirmed that succinylation at specific lysine sites (e.g., K1486) in metabolic enzymes, such as carboxyamide phosphate synthase 1 (CPS1) and hydroxymethylglutarate-CoA synthase 2 (HMGCS2), disrupts their substrate binding capacity, thereby leading to a loss of activity. However, the desamoylase SIRT5 has been shown to reverse this effect (60). In addition, the succinyl-CoA transferase OXCT1 has been identified as a catalyst for the succinylation of the mitochondrial protein LACTB, thereby promoting the progression of liver cancer through its disruptive effect on phospholipid metabolism. This finding indicates the active role of succinylation in metabolic reprogramming and tumourigenesis (23).

The interaction between succinylation and epigenetics is further manifested in the global regulatory effects triggered by metabolite accumulation. The presence of defects in SDH has been demonstrated to result in an abnormal accumulation of succinate. It has been demonstrated that, due to its structural similarity to *α*-ketoglutarate, succinate exerts a competitive inhibition effect on the activity of TET family DNA hydroxylases, resulting in genome-wide hypermethylation and histone hypermethylation, amongst other epigenetic dysregulations (61–63). Inhibition of SDH has been shown to increase the cellular sensitivity to energy stress, thus leading to metabolic plasticity and apoptotic susceptibility through glycolytic compensation of energy deficits (64, 65).

In conclusion, succinylation modification serves as a pivotal hub in the metabolic-epigenetic regulatory network, achieving multi-level integration through the following mechanisms: under physiological conditions, it maintains metabolic plasticity via dynamic equilibrium; under pathological conditions, abnormal succinylation drives disease progression by inducing metabolic reprogramming, triggering epigenetic disruption, and altering immune phenotypes. This regulatory property positions it as a core molecular bridge connecting energy sensing, gene expression regulation, and cell fate determination.

## 4 The pathological role of bidirectional regulatory networks in NDD

Neurodegenerative diseases are distinguished by a progressive loss of neurons in specific brain regions, which clinically manifests as cognitive decline, motor dysfunction, and memory impairment. The pathological processes in question are characterised by the interplay of multiple mechanisms, including, but not limited to, oxidative stress, mitochondrial dysfunction and neuroinflammation. Research indicates that the sirtuin family exerts significant neuroprotective functions in diseases such as AD, PD, amyotrophic lateral sclerosis (ALS), and multiple sclerosis (66).

Within the central nervous system, metabolic disorders have been observed to induce sustained bursts of mitochondrial reactive oxygen species (mtROS). Excessive ROS has been demonstrated to cause widespread oxidative damage to neuronal macromolecules, including proteins, lipids and nucleic acids. In addition to this, it also acts as a key signalling molecule. It has been demonstrated to profoundly reshape the epigenetic landscape by oxidatively modifying the active sites of epigenetic regulatory enzymes such as TET and HDAC, or by directly altering the chemical equilibrium of histone modifications. Conversely, epigenetic dysregulation exerts a further suppressive effect on the transcription of key antioxidant defence pathway genes, such as NRF2, thereby weakening neurons’ intrinsic capacity to counteract oxidative stress. This process initiates a self-reinforcing pathological cycle, “metabolic disorder → ROS burst → epigenetic dysregulation → metabolic gene suppression,” that continuously fuels the progression of neurodegenerative disease (67). It is important to note that this ROS-mediated vicious cycle also plays a central role in skeletal muscle degenerative diseases. Skeletal muscle, being a tissue with high metabolic demands, is highly dependent on mitochondrial aerobic metabolic efficiency for its function. In conditions of cellular senescence or pathological states, mitochondrial dysfunction within skeletal muscle tissues has been observed to induce a heightened production of mtROS. This has been shown to disrupt normal myocyte differentiation and regeneration by activating epigenetic mechanisms such as demethylation of key myogenic differentiation gene promoters and destabilising histone acetylation homeostasis of muscle-specific genes (68). Concurrently, ROS-mediated abnormal succinylation modifications may directly inhibit skeletal muscle glycolytic enzymes and contractile proteins, leading to insulin resistance, myofibrillar degradation, and sarcopenia. Collectively, these mechanisms suggest that the “neuro-muscular” regulatory axis, with ROS as a common node, may exert synergistic amplification effects in degenerative diseases (69).

Succinylation exhibits distinct subcellular localization patterns within the nervous system. Succinylated proteins have been found to accumulate predominantly in mitochondria and synaptic regions, where they participate in the regulation of energy metabolism, synaptic function and ferroptosis. The genes encoding these proteins are highly expressed in neurons, astrocytes, and endothelial cells, suggesting that succinylation has broad functional significance within the neurovascular unit (70). Piroli et al. (71) discovered that in a mouse model with impaired mitochondrial oxidative phosphorylation, fumarate-induced succinylation modification of dihydrolipoamide succinyltransferase (DLST) enhanced and inhibited *α*-KGDHC activity in specific brain regions affected by neurodegenerative lesions. As a pivotal rate-limiting enzyme within the TCA cycle, the purified KGDHC enzyme has the capacity to succinylate and modify the activity of numerous proteins. These include albumin and other enzymes that are integral to the TCA cycle. Its decreased activity leads to reduced succinyl-CoA production, which in turn exacerbates ATP deficiency. This biochemical deficit has been shown to induce metabolic dysfunction, leading to metabolic disorder in affected neurons.

**Figure 4:**
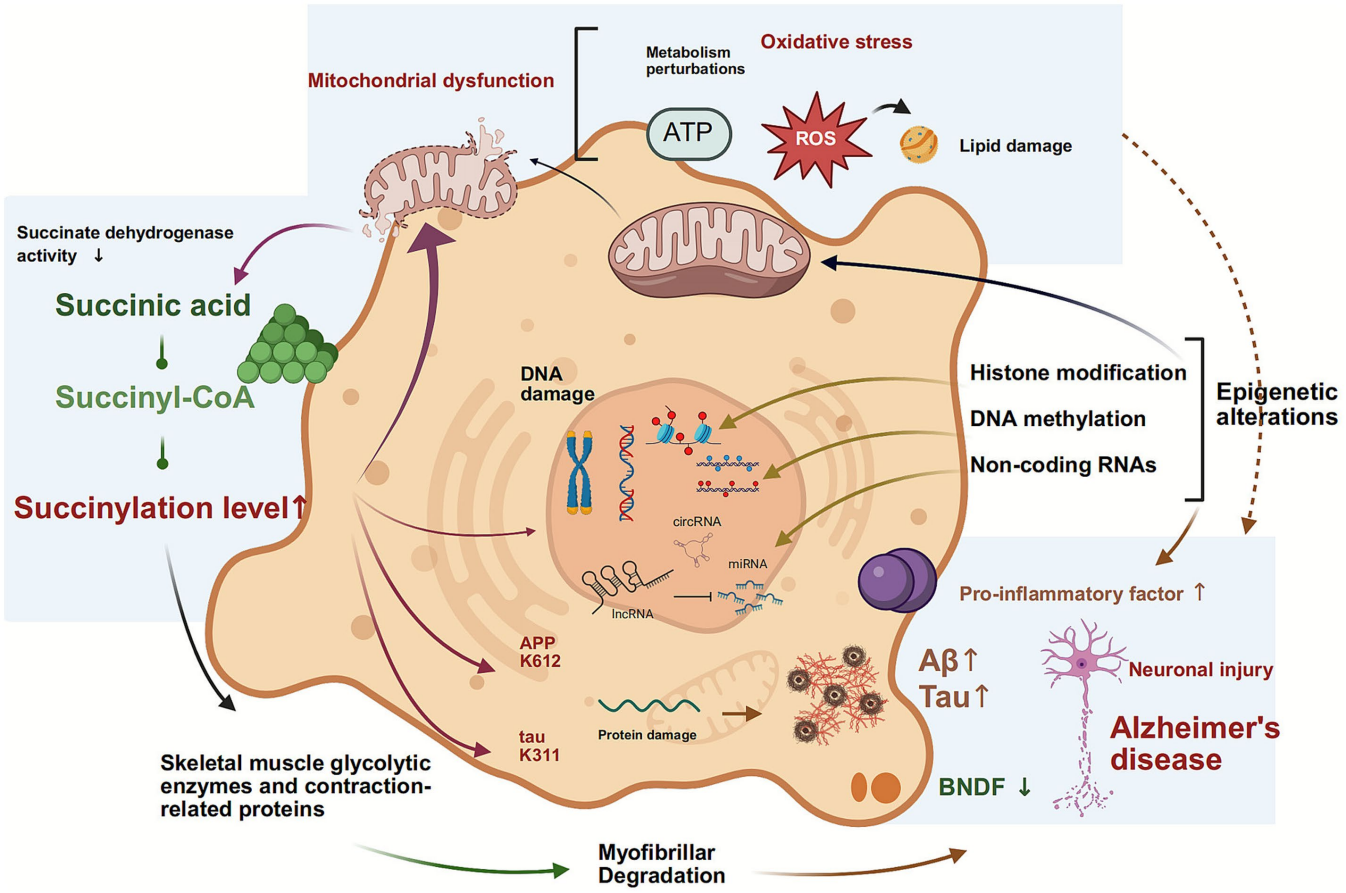
Lysine succinylation as a dynamic hub integrating metabolic and epigenetic hallmarks in Alzheimer’s disease. This schematic positions lysine succinylation as a critical mediator between core aging processes and AD pathogenesis. The model shows how metabolic inputs regulate succinylation, which then directly influences epigenetic programming. These epigenetic alterations subsequently form a feedback loop by repressing metabolic genes, thereby stabilizing a pathological state that exacerbates genomic instability, mitochondrial dysfunction, and the accumulation of Aβ and tau.

AD is typified by cognitive and memory impairment due to neuronal degeneration, primarily characterised by the presence of abnormal deposits of Aβ and tau proteins (72, 73). PTMs of tau protein constitute the major component of tau neurofibrillary tangles, which serve as pathological hallmarks of AD and other tauopathies (74). These modifications alter tau protein structure, affecting its interaction with microtubules, localization, degradation, and aggregation. Consequently, they influence its aggregation propensity, ultimately leading to neuronal damage and cognitive impairment (75). In the pathophysiology of AD, succinylation modification synergistically drives disease progression through dual pathways. Yang et al. (76) conducted a quantitative succinylation proteomics analysis and found that mitochondrial proteins in AD patient brain tissue exhibited broadly reduced succinylation levels, while specific succinylation increases were observed at the APP K612 and tau K311 sites. Mechanistic studies indicate that the succinylation of APP K612 disrupts normal proteolytic processing, promoting the production and oligomerization of Aβ and accelerating plaque formation. Concurrently, tau K311 succinylation significantly enhances aggregation propensity, facilitating the formation of neurofibrillary tangles and destabilising microtubule assembly (76). In pathological conditions, HDAC has been observed to upregulate the phosphorylation levels of glutamate, tau protein, and glial fibrillary acidic protein (GFAP), while concomitantly downregulating the expression of brain-derived neurotrophic factor (BDNF) (77). PTMs within the microtubule-binding domain (MBD) of tau, including two hexapeptide motifs, have been identified as key nucleation sites for tau aggregation. These modifications may also regulate tau aggregation and interactions with microtubules and membranes. Further studies are required to elucidate the molecular mechanisms by which succinylation affects tau protein function. Acosta et al. (78) utilised synthetic biology to demonstrate that succinylation at the K311 site within the PHF6 motif introduces a strong negative charge and steric hindrance, significantly disrupting the interaction between tau protein and the microtubule-binding domain (T2R complex), leading to a loss of microtubule stability. It is noteworthy that succinylation also modulates tau protein oligomerization kinetics and fibrillar assembly by altering its interaction patterns with lipid membranes, thereby influencing pathological aggregation at multiple levels (78). Collectively, these findings establish succinylation as a key regulator in core AD pathology: it promotes Aβ pathology by modulating APP processing while accelerating neurofibrillary tangle formation by altering tau’s microtubule-binding capacity and aggregation properties, constituting a crucial molecular basis for AD progression (Figure 4).

ALS and PD are severe neurodegenerative disorders characterised by progressive neuronal degeneration, for which no cure currently exists. The two diseases are characterised by protein aggregation, yet the specific proteins involved differ. ALS is primarily associated with TDP-43 and FUS, while PD is linked to *α*-synuclein. Research linking succinylation to ALS, PD, and other neurodegenerative diseases remains in its infancy. Chen et al. (79) investigated the PTM profiles of yeast models of ALS and PD. Their findings revealed a correlation between FUS aggregation and significant alterations in multiple histone marks, particularly H2B phosphorylation and H3 acetylation. The results of this study indicate that the over-expression of TDP-43 and *α*-synuclein caused a slight decrease in histone H3K36 methylation levels, suggesting the existence of interactions between histones. These findings suggest that epigenetic mechanisms may play a crucial role in the pathogenesis and progression of ALS and PD (79). As previously described, SIRT5, a major desamoylylase, exhibits activity dependent on NAD + levels. A decline in NAD + concentration has been evidenced to inhibit SIRT5 desamoylylation activity, resulting in elevated amoylation levels. These elevated amoylation levels have been demonstrated to impair metabolic enzyme activity and energy production. Research has found that expression of NMNAT2, a key protein in NAD + synthesis, is reduced in the spinal cord of ALS patients. This results in restricted SIRT5 activity, excessive succinylation of mitochondrial proteins, and consequently disrupts energy metabolism while promoting oxidative stress (80). Supplementing NAD^+^ precursors to restore SIRT5 desamoylation activity alleviates abnormal protein amoylation, thereby improving metabolic disorders and neurodegeneration—consistent with prior findings. NAD + metabolic disorders and mitochondrial dysfunction are also exhibited by neurodegenerative diseases such as PD, suggesting the NAD + –SIRT5–succinylation axis may also play a regulatory role.

From a metabolic perspective, Warburg-like metabolic reprogramming is significantly evident in neurodegenerative diseases (81). Research indicates that under physiological conditions, this effect supports synapse formation and neural plasticity by enhancing glycolysis to provide ATP and metabolic intermediates required for synaptic plasticity (58). However, in pathological conditions, succinylation-driven metabolic reprogramming disrupts this balance, resulting in insufficient ATP production, lactic acid accumulation, and the buildup of lipotoxic substances. This, in turn, has been demonstrated to induce sustained mitochondrial ROS production and chronic neuroinflammation. This metabolic-inflammatory axis imbalance is known to worsen with age, due to the onset of DNA damage and the promotion of protein misfolding. The result is a detrimental cycle involving impaired CNS energy supply, oxidative stress homeostasis collapse and chronic neuroinflammation, which significantly exacerbates neurodegeneration.

As a consequence of Vitamin B12 deficiency, there is a disruption of energy metabolism, epigenetic regulation, and lipid metabolism, due to the inhibition of succinyl-CoA production. SAM is a vital compound that facilitates methylation processes in DNA, RNA, proteins, neurotransmitters, and phospholipids. Depletion of SAM occurs as a result of methionine deficiency, which is often caused by insufficient intake of vitamin B12. This deficiency can further compound the damage to neural tissue. Furthermore, it has been established that methylation reactions that utilise SAM generate S-adenosylhomocysteine (SAH). In ordinary circumstances, SAH facilitates the recycling of adenosine and homocysteine through hydrolysis. However, it has been demonstrated that homocysteine (HCy) accumulation due to vitamin B12 deficiency can result in excessive SAH buildup, thereby inhibiting methyltransferase activity. These synergistic phenomena ultimately trigger significant molecular alterations, including DNA and histone epigenetic modifications, thereby affecting gene expression (82). Concurrently, reduced succinate levels may induce glycolytic gene transcription and release inhibition on histone/DNA demethylases, further amplifying epigenetic disruption (83, 84).

Fucans are sulfated polysaccharides that are extracted from brown algae. They are composed of L-fucose and sulfate groups, and they exhibit biological properties including antitumor, anti-inflammatory, antioxidant, and gut health regulation effects. Chen et al. (85) found cognitive impairment and neuronal morphological abnormalities in diabetic mouse models, while fucoidan supplementation elevated short-chain fatty acid (SCFA) levels in the cecum. These SCFAs enhanced TET2 protein stability by activating phosphorylated AMPK and increased TET2 activity by reducing the (succinate + malate)/ *α*-ketoglutarate ratio to enhance TET activity, thereby boosting TET2 function. Crucially, fucoidan prevented diabetic cognitive impairment by promoting TET2-mediated active DNA demethylation in the cerebral cortex of diabetic mice (85). This mechanism is homologous to the inhibition of TET by succinate/fumarate in the context of TET inhibition in tumours with SDH/FH defects. This phenomenon is facilitated by structural homology competition between succinate and *α*-KG for binding to the TET catalytic pocket, thereby unveiling a molecular coupling between metabolic dysregulation and DNA epigenetic regulation via imbalanced metabolite levels. The restoration of TET function activates neuroprotective gene expression, thereby mitigating cognitive impairment. Zhao et al. (86) established a SIRT5-knockdown BV2 microglial cell model and a lipopolysaccharide (LPS)-induced inflammation model. The combination of these with aged mouse and human brain samples resulted in the discovery that SIRT5 knockdown significantly elevated succinylation levels, thereby shifting microglial energy metabolism from oxidative phosphorylation towards glycolysis. This phenomenon was accompanied by an increase in mitochondrial ROS, lipid droplet accumulation, and upregulation of proinflammatory factors, including IL-1β, IL-6, and TNF-*α*. The study indicates that succinylation modification is associated with cellular energy metabolism reprogramming, manifested as impaired mitochondrial adaptability and a metabolic pathway shift toward glycolysis-dominant metabolism (86).

Succinylation modification has emerged as a pivotal player in the complex etiopathology of neurodegenerative diseases, exerting its regulatory influence over key pathological proteins, reprogramming cellular metabolism, and interacting with epigenetic networks. Intervention strategies targeting succinylation imbalance, including the modulation of NAD + levels, the targeting of desuccinylases, and the correction of metabolic defects, offer novel therapeutic perspectives for the slowing of the co-progression of neurodegenerative and muscular degeneration.

## 5 Conclusion

This review synthesizes recent advances in the interplay between metabolism and epigenetics, with a focus on the bridging role of lysine succinylation, and proposes a bidirectional succinylation regulatory network hypothesis. Evidence indicates that metabolic intermediates function not only as substrates in energy metabolism but also as direct regulators of epigenetic remodeling and neuronal function through succinylation modifications. Key succinylation targets are enriched in central metabolic pathways, including the TCA cycle, catabolic processes involving amino acids and fatty acids, and protein complexes governing energy transfer and ATP synthesis. Metabolic intermediates and end products can directly modulate protein activity or serve as acyl donors for post-translational and epigenetic modifications, thereby influencing neuronal repair processes and establishing a mechanistic link between cellular metabolism and neurological pathology. Aberrantly accumulated metabolites resulting from metabolic dysregulation covalently modify proteins, altering enzyme activity, protein conformation, and molecular interactions. These modifications further contribute to neurodegenerative disease progression by mediating epigenetic changes that affect brain plasticity.

Current research highlights the central role of succinylation in metabolic-epigenetic crosstalk in neurodegenerative diseases, however, multiple multidimensional questions remain unresolved. At the mechanistic level, the bidirectional regulation mediated by succinylation exhibits pronounced cell-type specificity and spatiotemporal dynamics. For instance, succinyl-CoA metabolism differs significantly between neurons and astrocytes: neurons depend on mitochondrial oxidative phosphorylation to meet high energy demands, rendering their succinylation levels particularly sensitive to TCA cycle fluctuations, whereas astrocytes support neuronal function via glycolysis, potentially coupling their succinylation patterns more closely to glycolytic flux. This cellular heterogeneity underscores the need to integrate single-cell sequencing with spatial proteomics to map cell-specific succinylation networks and their regulatory logic. From a translational standpoint, succinylation-targeted therapies should embrace a precision medicine framework. Future strategies may improve efficacy through cell-specific delivery systems—such as engineered lipid nanoparticles—or by designing peptide-based inhibitors selective for disease-associated succinylation sites.

Furthermore, as a molecular nexus connecting metabolism and epigenetics, the succinylation regulatory network may extend beyond the central nervous system to involve cross-organ communication, particularly with gut microbiota-derived metabolites. The succinate produced by gut microbiota has the capacity to affect the central nervous system via the bloodstream. Whether microbial dysbiosis promotes neurodegenerative disease progression by perturbing systemic succinate levels represents a compelling “gut-brain axis” pathway worthy of investigation.

In summary, the bidirectional succinylation regulatory network provides a novel conceptual framework for understanding the complex pathophysiology of neurodegenerative diseases. Future studies should adopt integrative, multidisciplinary approaches to advance understanding across molecular, cellular, and systems-level dimensions, ultimately enabling the development of early diagnostic biomarkers and targeted therapeutic interventions.
